# Baseline Susceptibility of Field Populations of *Helicoverpa armigera* to *Bacillus thuringiensis* Vip3Aa Toxin and Lack of Cross-Resistance between Vip3Aa and Cry Toxins

**DOI:** 10.3390/toxins9040127

**Published:** 2017-04-05

**Authors:** Yiyun Wei, Shuwen Wu, Yihua Yang, Yidong Wu

**Affiliations:** College of Plant Protection, Nanjing Agricultural University, Nanjing 210095, China; weiyiyun1988@163.com (Y.W.); swwu@njau.edu.cn (S.W.); yhyang@njau.edu.cn (Y.Y.)

**Keywords:** *Helicoverpa armigera*, Vip3Aa, Cry1Ac, Cry2Ab, susceptibility, cross-resistance

## Abstract

The cotton bollworm *Helicoverpa armigera* (Hübner) is one of the most damaging cotton pests worldwide. In China, control of this pest has been dependent on transgenic cotton producing a single *Bacillus thuringiensis* (Bt) protein Cry1Ac since 1997. A small, but significant, increase in *H. armigera* resistance to Cry1Ac was detected in field populations from Northern China. Since Vip3Aa has a different structure and mode of action than Cry proteins, Bt cotton pyramids containing Vip3Aa are considered as ideal successors of Cry1Ac cotton in China. In this study, baseline susceptibility of *H. armigera* to Vip3Aa was evaluated in geographic field populations collected in 2014 from major cotton-producing areas of China. The LC_50_ values of 12 field populations ranged from 0.053 to 1.311 μg/cm^2^, representing a 25-fold range of natural variation among populations. It is also demonstrated that four laboratory strains of *H. armigera* with high levels of resistance to Cry1Ac or Cry2Ab have no cross-resistance to Vip3Aa protein. The baseline susceptibility data established here will serve as a comparative reference for detection of field-evolved resistance to Vip3Aa in *H. armigera* after future deployment of Bt cotton pyramids in China.

## 1. Introduction

*Bacillus thuringiensis* (Bt) is a Gram-positive, soil-dwelling bacterium. Many Bt strains produce insecticidal crystal proteins (Cry proteins) during sporulation, as well as vegetative insecticidal proteins (Vips) during vegetative stages of growth [1,2,3]. Since 1996, some of these insecticidal Bt proteins (mainly Cry proteins) have been incorporated into transgenic crops (Bt crops) for control of several major pests of lepidoptera and coleoptera. In 2015, more than 84 million hectares of Bt cotton, corn, and soybean were planted globally [4]. Although Bt crops have provided effective control of target pests, reduced chemical insecticide use and increased profit, evolution of pest resistance to Bt proteins produced by transgenic crops is the main threat to the continued success of Bt crops [5,6]. The economic and environmental benefits of Bt crops have been lost because of rapid evolution of resistance by pests, particularly to the earliest commercialized Bt crops that produced only one Bt toxin [6,7].

To delay resistance, Bt crop pyramids producing two or more different Bt proteins that are effective against the same pest have become increasingly prevalent in the USA, India, Australia, and Brazil [7,8]. Pyramid strategy relies on the concept that insects resistant to one Bt protein will be killed by the other Bt protein, which is called “redundant killing” [9]. A key condition favoring durability of these pyramided crops is the absence of cross-resistance between Bt proteins [9,10,11,12].

The Vip insecticidal proteins (mostly refering to Vip3A) have no sequence similarity and share no binding sites compared with Cry proteins [1,13]. The distinct mode of action of Vip3A makes them good candidates to be pyramided with Cry proteins in transgenic Bt crops to delay insect resistance and to broaden the insecticidal spectrum [3].

*Helicoverpa armigera* (Hübner) is one of the most damaging agricultural pests in the world. This polyphagous pest is widespread throughout Europe, Africa, Asia, Australia, and recently invaded the New World [14]. In China, control of this pest has been dependent on Bt cotton producing a single Bt protein Cry1Ac since 1997. Due to intensive planting of Bt cotton, frequency of resistant individuals to Cry1Ac increased from 0.93% in 2010 to 5.5% in 2013 in field populations of *H. armigera* from Northern China [15,16]. Although non-Bt host crops serving as a natural refuge for *H. armigera* substantially delayed Cry1Ac resistance in field populations of *H. armigera* from Northern China [16], an immediate switch to dual Bt cotton (such as Cry1Ac + Cry2Ab) or three-toxin pyramided cotton (such as Cry1Ac + Cry2Ab + Vip3Aa) is suggested as an alternative tactics for mitigating Bt resistance in *H. armigera* [16,17,18].

In the present study, baseline susceptibility of *H. armigera* to Vip3Aa was evaluated in 12 geographic field populations collected in 2014 from major cotton planting areas of China. Toxicity of Vip3Aa to four laboratory strains with resistance to either Cry1Ac or Cry2Ab was also tested to assess the possible cross-resistance potential between Vip3Aa and Cry proteins.

## 2. Results

### 2.1. Susceptibility of Field Populations of H. armigera to Vip3Aa11

Toxicity data of Vip3Aa11 to 12 field populations and the susceptible SCD strain of *H. armigera* are shown in Table 1. The LC_50_ values of field populations ranged from 0.053 to 1.311 μg/cm^2^, representing a 25-fold range of variation among geographic populations.

The criterion of non-overlap of 95% fiducial limits was used to assess differences in LC_50_ between individual field populations and the susceptible SCD strain. The LC_50_ values did not differ significantly between eight of the 12 field populations and the susceptible SCD strain. The LC_50_ values were significantly greater for 3 of the 12 field populations than for SCD. Only one field population (Suzhou) had significantly lower LC_50_ value than SCD.

### 2.2. Susceptibility of Four Cry-Resistant Strains of H. armigera to Vip3Aa11

Bioassay results of Vip3Aa11 against four Cry-resistant strains and two susceptible laboratory strains (SCD and An) were shown in Table 2.

The LC_50_ values did not differ among SCD, SCD-r1, and SCD-A2KO1 based on overlap of 95% fiducial limits, showing that the two strains with recessive resistance to either Cry1Ac or Cry2Ab have no cross-resistance to Vip3Aa11. Similarly, the LC_50_ values were not significantly different among An, AY2, and An2Ab based on overlap of 95% fiducial limits, indicating that the two strains with dominant resistance to either Cry1Ac or Cry2Ab do not confer any cross-resistance to Vip3Aa11.

However, the LC_50_ values were significantly greater for the SCD, SCD-r1, and SCD-A2KO1 strains than for the An, AY2, and An2Ab strains. The SCD-r1 and SCD-A2KO1 strains are both derived from SCD, which was collected from Africa in the 1970s [19]. The An, AY2, and An2Ab strains are all derived from Anyang, Henan province of Northern China between 2009 and 2011 [18,20]. The difference in Vip3Aa11 susceptibility could represent the natural geographic variation between the two groups of strains.

## 3. Discussion

Establishment of baseline susceptibility data is usually completed prior to wide commercial adoption of a Bt crop, and the baseline data is necessary for defining susceptibility changes relating to exposure to Bt crops [21]. In the present study, baseline susceptibility of Vip3Aa11 was established from 12 geographical Chinese populations of *H. armigera*. These baseline data will serve as a comparative reference for early detection of field-evolved resistance after future deployment of Bt cotton pyramided with Vip3Aa in China.

Although Bt cotton producing Cry1Ac has been intensively planted in China since 1997, there is no evidence showing any correlation between responses to Cry1Ac and Vip3Aa [17]. In addition, our bioassay results demonstrated that several strains of *H. armigera* with resistance to either Cry1Ac or Cry2Ab have no cross-resistance to Vip3Aa11. Therefore, the susceptibility difference to Vip3Aa11 (25-fold variation at LC_50_) among 12 *H. armigera* populations from China represents the natural geographic variation, and is not related to previous exposure to Bt cotton producing Cry1Ac.

The risk of rapid pest adaptation to an insecticide or a Bt protein is highly dependent on the initial frequency of resistance alleles in field populations [5,21]. Before deployment of Bt cotton expressing Vip3Aa in Australia, baseline frequencies of Vip3Aa resistance alleles were determined in Australian populations of *H. armigera* and *H. punctigera* with the F_2_ screen method [22]. The genotypic screen results showed that relatively high frequency of Vip3Aa resistance alleles exists in field populations of both *Helicoverpa* species (0.027 for *H. armigera*, 0.008 for *H. punctigera*) [22]. In contrast, relatively low frequency of Vip3Aa resistance alleles (about 0.001) was estimated with the F_2_ screen method in Brazilian populations of *Spodoptera frugiperda* [23]. It will be of special interest to investigate the frequency and diversity of Vip3Aa resistance alleles in field populations of *H. armigera* from China.

Vip3Aa is an exotoxin produced and secreted during the vegetative growth stage of Bt, whereas Cry proteins are endotoxins produced during sporulation. Vip3Aa shares no sequence homology with any known Bt Cry proteins. Further studies showed that Vip3Aa and Cry proteins differ in several key steps necessary for insecticidal activity [3,24]. So far, a number of investigations have confirmed cross-resistance is nonexistent between Vip3Aa and Cry proteins. Jackson et al. (2007) found three *Heliothis virescens* strains with variable levels of resistance to Cry1Ac and/or Cry2Ab are equally susceptible to the Vip3Aa protein [25]. The current study also confirmed that four *H. armigera* strains, with high levels of resistance to Cry1Ac or Cry2Ab and a diverse genetic basis of resistance, have no cross-resistance to Vip3Aa protein. Recently, a study demonstrated that several Vip3Aa resistant strains of *H. armigera* and *H. punctigera* were not cross-resistant to Cry1Ac or Cry2Ab [22]. Thus, it is expected that Vip3Aa will favorably complement Bt Cry proteins in pyramided crops for increasing the sustainability of Bt technologies.

Cotton farmers in China still plant the first generation Bt cotton that produces only Cry1Ac, whereas farmers in Australia, the United States, and India have switched to the second generation Bt cotton which produces both Cry1Ac and Cry2Ab [7,16]. Bt cotton producing Cry1Ac, Cry2Ab, and Vip3Aa will be commercially deployed in Australia in the 2016/17 cotton season [26]. Considering the current status of Cry1Ac resistance and cross-resistance risk between Cry1Ac and Cry2Ab [15,16,18,20], replacement of the first generation Bt cotton with the three-toxin pyramids could be more effective and more durable for delaying Bt resistance evolution of *H. armigera* in China.

## 4. Materials and Methods 

### 4.1. Insect Strains and Rearing

Field populations of *H. armigera* in the study were collected during June to August of 2014 from major cotton planting areas of China (Table 3). Colonies were established from collections (adults or eggs) made on cotton plants located across 12 provinces of China. We collected male and female moths by light trap in most sites except Yancheng, Suzhou, and Shawan, where eggs on Bt cotton plants were collected. Insects from the collected eggs were reared to adults in the laboratory on an artificial diet. We tested the F_1_ progeny from all sites with bioassays as described below.

The susceptible SCD strain of *H. armigera* originated from the Ivory Coast, Africa in the 1970s and was passed to our laboratory by Bayer Crop Science in 2001. The SCD strain was maintained in the laboratory without exposure to insecticides or Bt toxins [19]. The SCD strain was used to check the consistency of bioassays across years throughout the duration of the study. Another susceptible strain of *H. armigera*, called strain An, was started in June 2009 from the progeny of more than 100 field-mated females collected in Anyang, Henan province of Northern China. The An strain has been maintained in the laboratory without exposure to insecticides or Bt toxins since collection.

Resistance characteristics of four Cry-resistant strains of *H. armigera* were detailed in Table 4. SCD-r1 strain was created by introgressing the *r1* allele of the cadherin gene (*HaCad*) from the GYBT strain into the susceptible SCD strain [19]. The ABC transporter gene *HaABCA2* of the SCD strain was knocked out with CRISPR/Cas9 system, and the knockout strain was made homozygous and named SCD-A2KO1 (unpublished data). AY2 strain with dominant resistance to Cry1Ac was isolated from a field population collected during June 2011 from Anyang, Henan province of China [20]. An2Ab strain was selected with Cry2Ab from a field population of *H. armigera* collected in June 2009 from Anyang, Henan province of China [18].

Larvae of *H. armigera* were reared on an artificial diet based on wheat germ and soybean powder at 27 °C (±1 °C) with a 16 h L:8 h D photoperiod. Adults were held under the same temperature and light conditions at 60% (±10%) RH and supplied with a 10% sugar solution.

### 4.2. Vip3Aa Protein

The Vip3Aa11 protoxin was provided by the Institute of Plant Protection; Chinese Academy of Agricultural Sciences (CAAS); Beijing; China. The plasmid (pET-3Aa11) harboring the *Vip3Aa11* gene (NCBI accession No. JN226104.1) was transformed into *Escherichia coli* (BL21); which was used as a source of toxin. The *Vip3Aa11* gene was modified to contain a His tag sequence at the C terminus of the Vip protein to facilitate purification. Expression of Vip3Aa11 protein was induced with isopropyl-β-d-thiogalactopyranoside (IPTG); and cells were broken by sonication treatment. Vip3Aa11 protein from cell lysates was purified on a HiTrap Chelating HP column (GE Healthcare, Freiburg, Germany) and was examined for purity by sodium dodecyl sulfate-polyacrylamide gel electrophoresis (SDS-PAGE) analysis. The Vip3Aa11 protein in the crude cell lysate was quantified by densitometry of the stained band (~88 kDa) on the SDS-PAGE gel. The crude cell lysate was stored at −80 °C until use for bioassays.

### 4.3. Bioassays

We used diet surface overlay bioassay, which is similar to the method established in Australia for testing Vip3Aa toxicity against *H. armigera* [22]. Toxin stock suspensions were diluted with 0.01 M, pH 7.4 phosphate-buffered solution (PBS). Liquid artificial diet (1000 μL) was dispensed into each well of a 24-well plate. After the diet cooled and solidified, 100 μL of the toxin solution was applied evenly to the diet surface in each well and allowed to air dry, and a single neonate (less than 24 h old) was placed in each well. Forty-eight neonates were tested for each concentration of Vip3Aa11, including a control with only PBS. All tests were kept at 26 °C (± 1 °C) and 60% (± 10%) RH with a photoperiod of 16 h L:8 h D. After seven days, we scored larvae as dead if they died or if they weighed less than 5 mg.

### 4.4. Data Analysis

We used the Poloplus program (2002, Version 1.0, Berkeley, CA, USA) [27] to conduct probit analysis of the concentration-mortality data to estimate the concentration killing 50% of larvae tested (LC_50_), the 95% fiducial limits of the LC_50_, the slope of the concentration-mortality line and the standard error of the slope. We considered two LC_50_ values significantly different only if their 95% fiducial limits did not overlap [28].

## Figures and Tables

**Table 1:** Concentration-mortality responses to Vip3Aa11 insecticidal protein of the laboratory susceptible SCD strain and 12 field populations of *Helicoverpa armigera* collected in 2014.

Population	*n* ^1^	Slope ± SE	LC_50_ (µg/cm^2^)	95% FL ^2^	χ^2^	d*f* ^3^	TR ^4^
SCD	226	1.62 ± 0.20	0.194	0.131–0.293	5.798	5	3.7
Shawan	295	1.19 ± 0.14	0.372	0.150–1.034	18.759	5	7.0
Anci	217	1.63 ± 0.23	0.227	0.120–0.731	10.431	4	4.3
Gaoyang	217	2.19 ± 0.28	0.358	0.278–0.455	3.862	4	6.8
Cangxian	215	1.47 ± 0.18	1.032	0.498–2.017	11.729	5	19.5
Nanpi	237	1.35 ± 0.18	0.507	0.263–0.939	10.043	5	9.6
Qiuxian	232	2.48 ± 0.27	0.199	0.136–0.312	7.371	4	3.8
Huimin	251	1.58 ± 0.20	0.176	0.047–0.406	16.362	4	3.3
Xiajin	204	1.91 ± 0.24	1.311	1.027–1.738	1.244	4	24.7
Anyang	222	1.69 ± 0.22	0.404	0.218–0.788	9.099	4	7.6
Kaifeng	209	2.46 ± 0.28	0.442	0.315–0.618	4.956	4	8.3
Yancheng	273	1.42 ± 0.19	0.508	0.160–0.950	9.276	4	9.6
Suzhou	216	3.02 ± 0.41	0.053	0.042–0.064	2.256	3	1.0

^1^ Total number of individuals tested; ^2^ 95% fiducial limits of LC_50_; ^3^ Degree of freedom; ^4^ TR (tolerance ratio) = LC_50_/LC_50_ of the Suzhou population.

**Table 2:** Concentration-mortality responses to Vip3Aa11 insecticidal protein of two susceptible laboratory strains (SCD and An) of *Helicoverpa armigera* and four laboratory strains resistant to Bt Cry1Ac or Cry2Ab toxins.

Strain ^1^	*n* ^2^	Slope ± SE	LC_50_ (µg/cm^2^)	95% FL ^3^	χ^2^	d*f* ^4^	TR ^5^
SCD (s)	592	2.32 ± 0.34	0.111	0.059–0.163	8.249	4	3.8
SCD-r1 (r, Cry1Ac)	295	2.04 ± 0.30	0.108	0.056–0.160	6.555	5	3.7
SCD-A2KO1 (r, Cry2Ab)	316	1.63 ± 0.16	0.086	0.056–0.129	8.608	5	3.0
An (s)	193	2.61 ± 0.40	0.029	0.011–0.047	4.589	3	1.0
AY2 (d, Cry1Ac)	242	1.98 ± 0.24	0.029	0.018–0.040	4.439	4	1.0
An2Ab (d, Cry2Ab)	596	2.09 ± 0.18	0.040	0.027–0.053	4.384	3	1.4

^1^ r: recessive resistance; d: dominant resistance; s: susceptible; ^2^ Total number of individuals tested; ^3^ 95% fiducial limits of LC_50_; ^4^ Degree of freedom; ^5^ TR (tolerance ratio) = LC_50_/LC_50_ of the strain An.

**Table 3:** Sampling information of *Helicoverpa armigera* field populations collected from China during June to August of 2014.

Population	Location	Latitude, Longitude	Stage at Collection	No. of Insects Collected
Shawan	Shawan, Xinjiang	44.33° N, 85.62° E	Egg	350
Anci	Langfang, Hebei	39.52° N, 116.68° E	Moth	630
Gaoyang	Baoding, Hebei	38.68° N, 115.78° E	Moth	181
Cangxian	Cangzhou, Hebei	38.30° N, 116.87° E	Moth	131
Nanpi	Cangzhou, Hebei	38.03° N, 116.70° E	Moth	179
Qiuxian	Handan, Hebei	36.82° N, 115.17° E	Moth	413
Huimin	Binzhou, Shandong	37.48° N, 117.50° E	Moth	170
Xiajin	Dezhou, Shandong	36.95° N, 116.00° E	Moth	205
Anyang	Anyang, Henan	36.10° N, 114.38° E	Moth	630
Kaifeng	Kaifeng, Henan	34.80° N, 114.30° E	Moth	252
Yancheng	Yancheng, Jiangsu	33.35° N, 120.15° E	Egg	300
Suzhou	Suzhou, Anhui	33.63° N, 116.98° E	Egg	360

**Table 4:** Resistance characteristics of four Cry-resistant strains of *Helicoverpa armigera*.

Strain	Bt Toxin Resisted	Resistance Fold at LC_50_	Dominance of Resistance	Resistance Mechanism	Reference
SCD-r1	Cry1Ac	440	Recessive	*HaCad* truncated	[19]
SCD-A2KO1	Cry2Ab	>100	Recessive	*HaABCA2* knocked out by CRISPR/Cas9 system	Unpublished
AY2	Cry1Ac	1200	Dominant	To be identified	[20]
An2Ab	Cry2Ab	130	Dominant	To be identified	[18]
